# A Systemic Review and Meta-analysis of the Leading Pathogens Causing Neonatal Sepsis in Developing Countries

**DOI:** 10.1155/2021/6626983

**Published:** 2021-06-05

**Authors:** Desalegne Amare Zelellw, Getenet Dessie, Endalkachew Worku Mengesha, Melashu Balew Shiferaw, Masresha Mela Merhaba, Solomon Emishaw

**Affiliations:** ^1^Department of Pediatrics and Child Health Nursing, College of Medicine and Health Sciences, Bahir Dar University, Bahir Dar, Ethiopia; ^2^Deparement of Adult Health Nursing, College of Medicine and Health Sciences, Bahir Dar University, Bahir Dar, Ethiopia; ^3^Department of Reproductive Health and Population Studies, College of Medicine and Health Sciences, Bahir Dar University, Bahir Dar, Ethiopia; ^4^Amhara Public Health Institute, Bahir Dar, Ethiopia; ^5^Amhara Regional Health Bureau, Bahir Dar, Ethiopia; ^6^Department of Emergency and Critical Care Nursing, College of Medicine and Health Sciences, Bahir Dar University, Bahir Dar, Ethiopia

## Abstract

**Background:**

Neonatal sepsis is one of the major public health problems globally, particularly, in developing countries. *Klebsiella*, *Staphylococcus aureus*, *Coagulase-negative Staphylococcus*, and *Escherichia coli* are the common pathogens for neonatal sepsis in developing countries. However, the pooled estimate of common pathogens causing neonatal sepsis in developing countries is still unknown. Therefore, this study is aimed at computing the pooled proportion of the leading cause of pathogens for neonatal sepsis in developing countries.

**Methods:**

We strictly followed the Preferred Reporting Items for Systemic Reviews and Meta-analysis guidelines to report this systematic review and meta-analysis. PubMed, Cochrane Library, Web of Science, CINAHL, Science Direct, and other search engines such as Google Scholar, Africa Journals Online, and Hinari were used to obtain studies related to the leading cause of pathogens for neonatal sepsis in developing countries. The search was done from October 1 to December 30, 2018, by considering both published and gray literature. Studies were evaluated based on the PRISMA guideline checklist by using their titles, abstracts, and full texts. Studies were extracted using Microsoft Excel spreadsheets, and STATA software version 14 was used to analyze data. Heterogeneity between studies was checked based on Cochran's *Q*-test and the corresponding *I*^2^ statistic test.

**Results:**

The pooled prevalence of the leading cause of pathogens of neonatal sepsis in developing countries were *Klebsiella* (26.36%), *Staphylococcus aureus* (23.22%), *Coagulase-negative Staphylococcus* (23.22%), and *Escherichia coli* (15.30%). Common pathogens were varied across regions; for instance, pooled isolated *Coagulase-negative Staphylococcus* was 25.98% in Africa, 16.62% in Asia, and 36.71% in Latin America, and *Klebsiella* was 29.80% in Africa, 23.21% in Asia, and 22.00% in Latin America. Also, *Staphylococcus aureus* was 27.87% in Africa and 18.28% in Asia, and *Escherichia coli* was 22.97% in Asia and 9.43% in Africa.

**Conclusions:**

This study highlights that the more prevalent common isolated pathogens in developing countries were *Klebsiella*, *Staphylococcus aureus*, *Coagulase-negative Staphylococcus*, and *Escherichia coli*, *Klebsiella*, and *Staphylococcus aureus* pathogens were predominantly high in Africa as compared to other Asian and Latin American countries. At the same time, *Coagulase-negative Staphylococcus* was more prevalent in Latin America compared to other regions. *Escherichia coli* is more dominant in Asia as compared to Africa and Latin America.

## 1. Background

Despite the lack of consensuses in definitions and variability between regions, neonatal sepsis is defined as a clinical syndrome of bacteremia with systemic signs and symptoms of infection in the first four weeks of life [1–5].

Neonatal sepsis is a major cause of mortality and morbidity in developing countries [6]. An estimated 3 million newborns suffer from sepsis globally every year [7]. A report showed that three out of every ten deaths were due to neonatal sepsis [8]. Globally, 15% of neonatal mortality was related to sepsis in 2016 [9, 10]. From the total mortality, nearly about 1.6 million deaths occur due to neonatal infections worldwide, and 40% of this death was found in developing countries [11]. Neonatal sepsis remains a significant global problem with little progress made despite major efforts [5] especially in developing countries [12]. This causes an annual economic burden ranging from $10 billion to $469 billion in sub-Saharan countries [13].

The common pathogens of early-onset neonatal sepsis in developed countries were *Group B Streptococcus* (43-58%), *Escherichia coli* (*E. coli*) (18-29%), and other gram-negative bacteria (7-8%). Similarly, in late-onset neonatal sepsis, the common pathogens were *Coagulase-negative Staphylococcus* (39-54%), *E. coli* (5-13%), *Staphylococcus aureus* (6-18%), and *Klebsiella* (4-9%) [14–16]. Hospital-acquired common pathogens of neonatal sepsis in developing countries were *Klebsiella* (16-28%), *Coagulase-negative Staphylococcus* (8-28%), *Staphylococcus aureus* (8-22%), and *E. coli* (5-16%). Also, community-acquired common pathogens of neonatal sepsis were *Staphylococcus aureus*, *Klebsiella*, *E. coli*, and *Group B Streptococcus* which accounted for 13-26%, 14-21%, 8-18%, and 2-8%, respectively [17–19].

*E. coli* is identified as the second leading cause of early-onset neonatal sepsis and accounted for about 24% of early-onset neonatal sepsis episodes and most (81%) infection seen in preterm newborn babies [20]. In very low birth weight babies, *E. coli* is responsible for 33.4% of the cases of early-onset neonatal sepsis [21, 22]. Similarly, *Staphylococcus aureus* and *CoNS* are more frequent causes of late-onset neonatal sepsis particularly in very low birth weight infants. Also, *CoNS* was commonly associated with neonatal sepsis in preterm infants, which accounts for 60 to 93% of bloodstream infections [23, 24].

International experience showed that the Gram-positive and Gram-negative microorganisms accounted for 44.5% *of Staphylococcus aureus*, 31.3% for other staphylococci, and 9.3% for *E. coli* [25]. Similarly, in developing countries, early-onset neonatal sepsis (EONS) is usually caused by Gram-negative pathogens, i.e., *E. coli* and *Klebsiella*, while late-onset neonatal sepsis is mainly caused by Gram-positive organisms like *CoNS*, *Staphylococcus aureus*, and *S. pneumonia*, although the percentage of late-onset sepsis caused by Gram-negative organisms are increasing [26–28]. *Klebsiella* and *E. coli*, in particular, are responsible for 61% of neonatal infections, and staphylococci are the most common Gram-positive bacteria for neonatal infection [29].

Moreover, *Klebsiella*, *Staphylococcus aureus*, *E. coli*, *Group B Streptococcus*, *S. pneumonia*, and *Salmonella* sp. have a major contribution for community-acquired neonatal sepsis, whereas hospital-acquired neonatal sepsis caused by *Klebsiella*, *Staphylococcus aureus*, *E. coli*, *CoNS*, *Pseudomonas* sp., *Enterobacter* sp., and *Candida* sp. were the common pathogens [12]. *Staphylococcus aureus*, *E. coli*, and *Klebsiella* are also the major causes of neonatal sepsis in developing countries [18].

Based on the acquisition of infection, neonatal sepsis can be classified as hospital acquired or community acquired [18, 30]. Hospital-acquired neonatal infection is the most common and severe infection among neonates hospitalized in the hospital [31, 32]. Although there is uncertainty on the source of infection, whether “maternally acquired” or “hospital acquired,” any infection associated with birth in a hospital is considered a hospital-acquired neonatal infection [17]. On the other hand, community-acquired neonatal sepsis is defined as “an infection occurring in nonhospitalized infants between the age of 7-90 days with ≥1 positive blood or CSF cultures with a recognized blood pathogen.” A new infection had to be separated by 48 hours from prior hospitalization discharge [30].

Despite the high burden of neonatal sepsis observed worldwide, there is no clear evidence on the rank of common pathogens leading to neonatal sepsis particularly in developing countries [33]. Gaps were identified on the current knowledge of common pathogens causing neonatal sepsis in low-income countries [34]. Current evidence on the leading cause of neonatal sepsis is varied across developing countries [35]; this may be due to the presence of heterogeneous population and healthcare settings [36]. Based on these regional variations, reporting a pooled analysis stratified by region is essential to synthesize recent evidence. Therefore, the ultimate aim of this systematic review and meta-analysis was to generate updated evidence on common pathogens causing neonatal sepsis in developing countries and to compute a single estimated proportion of common pathogens causing neonatal sepsis in developing countries. This may be important to choose appropriate antibiotics as an empirical treatment in low-income settings

## 2. Methods

### 2.1. Eligibility Criteria

We restricted our search to studies published in English. Obtained studies that cover pathogens causing neonatal sepsis in developing countries were carefully assessed whether they fulfilled our criteria or not. We only included observational studies that had full text and information on neonatal sepsis caused by common pathogens such as *Klebsiella* and/or *Coagulase-negative Staphylococcus* and/or *Staphylococcus aureus* and/or *E. coli* and neonatal sepsis diagnosed according to standard laboratory methods, i.e., blood culture, and supported with clinical presentations to diagnose *Coagulase-negative Staphylococcus* because of false-positive blood cultures due to contamination. The gold standard for diagnosis of neonatal sepsis, however, remains blood culture [37, 38]. In this study, we included studies from developing countries. For this study, we defined developing countries as “countries in the process of change with economic growth that increases in production, per capita consumption, and income.” Studies were excluded if the age of the study population was beyond the neonatal period (28-day-old infants) and had low sample sizes of less than 60 subjects. Also, we excluded studies that have methodological problems and flaws (lack of clear measurement, incomplete diagnostic criteria in choosing, selection bias, and unclear presentation of study population). Studies with a case-control study design were also excluded from the study.

### 2.2. Information Sources

Electronic databases and search engines were used to gather data about common pathogens of neonatal sepsis. The search was done from October 1 to December 30, 2018, which considered both published and gray literature.

### 2.3. Search Strategy

We strictly followed the Preferred Reporting Items for Systemic Reviews and Meta-analysis (PRISMA) flow diagram [39] to report this study. International electronic databases such as PubMed, Cochrane Library, Web of Science, CINAHL, Science Direct, and other searching engines such as Google Scholar, Africa Journals Online, and Hinari Access to Research for Health program were used to obtain studies related to the leading cause of pathogens for neonatal sepsis in developing countries. Our search protocol was developed using the following keywords: neonatal, neonatal sepsis, sepsis, *Klebsiella*, *CoNS*, *Staphylococcus aureus*, developing countries, developing, countries, developing nations, less developed nations, Africa, Latin America, and Asia. These terms were predefined to have an inclusive search strategy that involved all fields within records and searching medical literature using medical subject headings in the National Library of Medicine to control the vocabulary that indexed articles from the MEDLINE and/or PubMed database (Sup. File).

### 2.4. Study Selection and Data Extraction

Articles identified by the search were imported into EndNote version 7 to screen for duplication. Initially, the studies were selected by DAZ using their titles based on predefined inclusion and exclusion criteria. The coauthors (GD, EW, and MB) checked the consistency of the selected articles. The disparities between these reviewers were resolved by the other coauthors (SE and MM) through discussions. In the second phase, DAZ, GD, and EW screened articles using their abstracts. In the third phase, full-text articles were screened by DAZ and GD. The disagreements among reviewers during the selection process were resolved by discussion with MB, SE, and MM. Finally, all reviewed studies that fulfill the inclusion criteria were saved.

Studies were extracted by considering the preferred reporting items for systematic reviews and meta-analyses guidelines [40] and evaluated based on the PRISMA guideline. Duplicate studies were removed on the first screening process. Then, titles were carefully assessed and articles irrelevant to our objective were removed from the study. The authors DAZ, GD, and EW extracted data using Microsoft Excel spreadsheets. The extracted data comprised of authors' names, publication years, sites (institution or community), types of study (cross-sectional, retrospective), and total sample sizes. Discrepancies among reviewers were resolved by discussion and consensus with reviewers (MM, MB, and SE).

### 2.5. Quality of the Study

The quality of studies was approved using the Newcastle-Ottawa Scale (NOS) [41]. The NOS is designed to evaluate the qualitative evaluation of observational studies. This examines each study by seven items in three groups: selection, comparability, and outcome. Stars were given to each item. Items of good quality received 3 or 4 stars in the selection domain, 1 or 2 stars in the comparability domain, and 2 or 3 stars in the outcome/exposure domain. Items of fair quality received 2 stars in the selection domain, 1 or 2 stars in the comparability domain, and 2 or 3 stars in the outcome/exposure domain. Items of poor quality received 0 or 1 star in the selection domain, 0 stars in the comparability domain, and 0 or 1 stars in the outcome/exposure domain. In general, each item was scored for a maximum of six scores. Publications which scored 0–2, 3, 4, and 5 were classified as “unsatisfactory,” “satisfactory,” “good,” and “very good,” respectively. Finally, studies that had been categorized as low quality were excluded in the study [41].

### 2.6. Outcome of Interest

The outcome of interest was to determine the leading cause of pathogens (*Klebsiella*, *CoNS*, *Staphylococcus aureus*, and *E. coli*) for neonatal sepsis in developing countries. The pooled isolated common pathogens were measured as the number of neonates with sepsis caused by each respective pathogen divided by the number of neonates in a study multiplied by 100.

### 2.7. Statistical Analysis

Microsoft Excel spreadsheet was used to extract data, and STATA software version 14 was used to analyze data. Laird's random effects model was used to estimate the pooled proportion of common isolated pathogens for neonatal sepsis because a high degree of heterogeneity was observed across studies. Metaregression was employed to identify the source of heterogeneity using the year of publication, study design, and setting, but there was no statistically significant variable. Subgroup analysis was done by study setting to minimize the random variations between the point estimates of the primary studies. A funnel plot was applied to identify the presence of publication bias. Also, we employed Egger's test and Begg's statistical test to identify publication bias. Then, the trim and fill analysis was done to approve the presence of publication bias. A sensitivity analysis was performed to investigate how each study affects the estimated pooled prevalence. Statistical tests were significant if the *P* value was *<*0.05.

### 2.8. Publication Bias, Heterogeneity, and Sensitivity Test

Heterogeneity among studies was checked based on Cochran's *Q*-test and the corresponding *I*^2^ statistic test [42]. Continuous and categorical metaregression analyses were done to determine the sources of heterogeneity. Begg's test and Egger's test were also used to evaluate the publication bias [43].

## 3. Results

### 3.1. Description of the Studies

**Figure 1:**
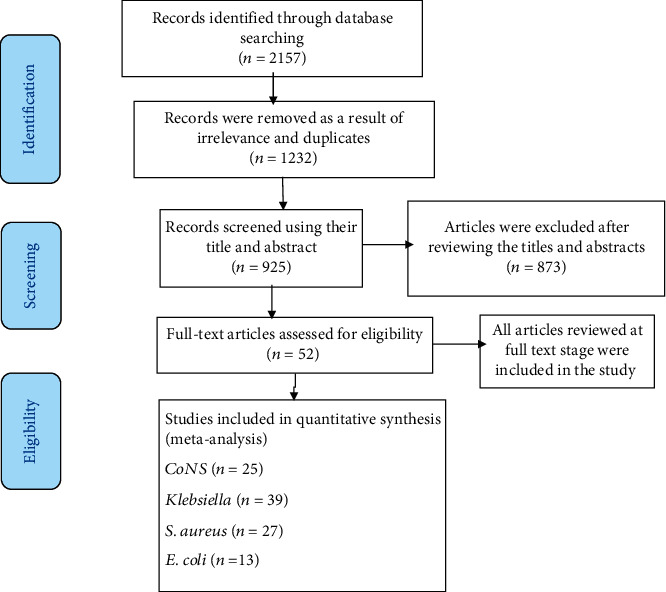
The PRISMA chart to report systematic reviews and meta-analyses of studies.

In the initial search, a total of 2157 potentially relevant studies were identified by searching international electronic databases, and 1232 studies were removed as a result of irrelevance and duplicates. Then, 925 studies were assessed in depth, and finally, a total of 52 studies were eligible for all common pathogens (25 studies for *CoNS*, 39 studies for *Klebsiella*, 13 studies for *E. coli*, and 27 studies for *Staphylococcus aureus*) (Figure 1) and 152217 infants were eligible for the final systematic review and meta-analysis. Studies involved in this systematic review and meta-analysis included 21 studies from different countries in developing regions. About 21 studies were from Africa, 4 studies were from Latin America, and 12 studies were from Asian countries. The publication year included in this study was from 2005 to 2018; the individual study with the largest sample size had 34362 infants, and the individual study with the smallest sample size had 68 infants.

### 3.2. Study Characteristics

#### 3.2.1. *Coagulase-Negative Staphylococcus*

In this study, twenty-five studies were included. All studies were conducted in hospitals. There were eleven studies each from Africa and Asia. The remaining three studies were conducted in Latin America. The largest sample size is 11,790, and the smallest sample size is 68 (Table 1).

#### 3.2.2. Klebsiella

Thirty-nine studies were included, and there were eighteen studies each from Africa and Asia. Three studies were included from Latin America. One study was conducted in a health center, and one study was conducted in a community. The remaining 37 studies were conducted in hospitals. Of the total, 18 studies were conducted using a cross-sectional study design and 21 studies were conducted using a cohort study design. The largest sample had 34362 infants, and the smallest size had 75 infants (Table 2).

#### 3.2.3. Staphylococcus aureus

A total of twenty-seven studies on the *Staphylococcus aureus* pathogen were included. Thirteen studies were conducted in Asia, and fourteen studies were conducted in Africa. Two studies were conducted in health centers, and one study was conducted in a community. Fourteen studies were conducted using a cohort study design (Table 3).

#### 3.2.4. Escherichia coli

Thirteen studies were included for the *E. coli* pathogen. All studies were conducted in hospitals. Majority (10/13) of the studies were conducted using a cross-sectional study design (Table 4).

### 3.3. Leading Pathogens of Neonatal Sepsis

**Figure 2:**
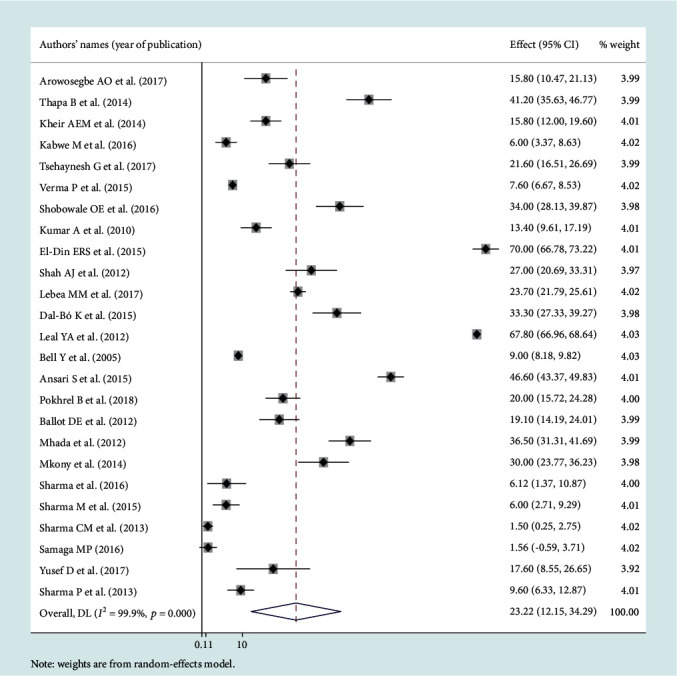
Forest plot for coagulase-negative Staphylococcus pathogen.

**Figure 3:**
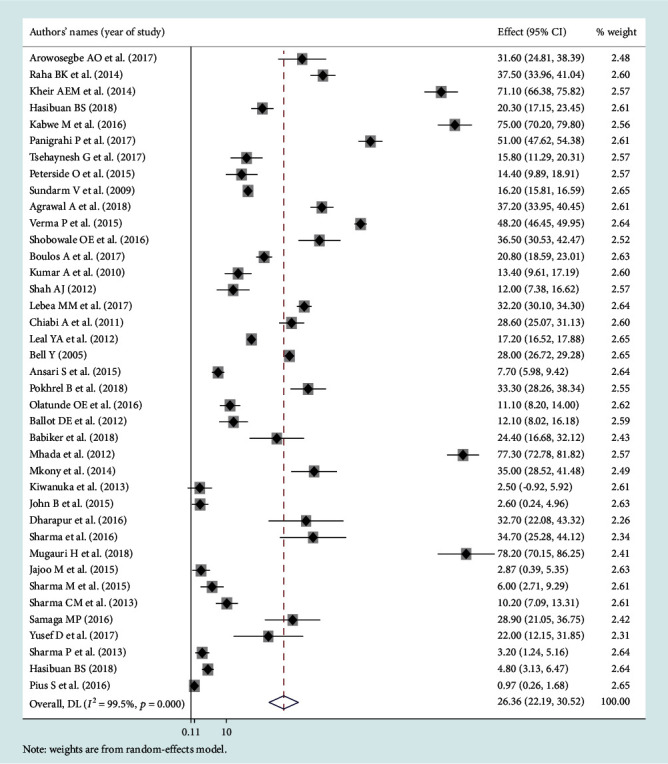
Forest plot for *Klebsiella* pathogen.

**Figure 4:**
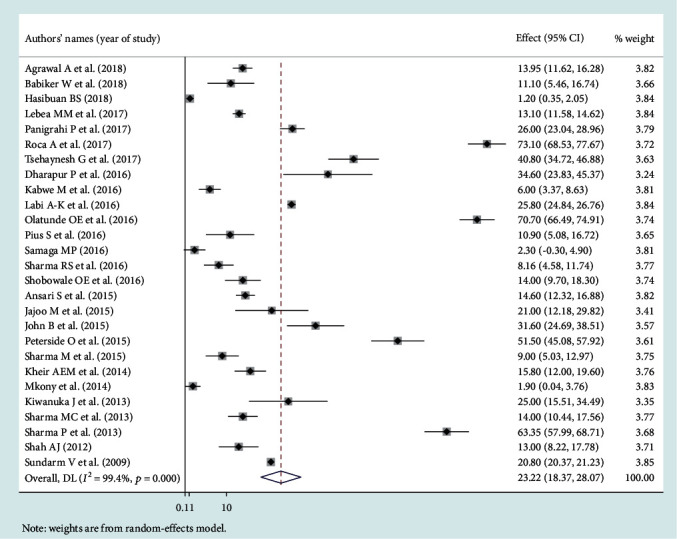
Forest plot for *Staphylococcus aureus* pathogen.

**Figure 5:**
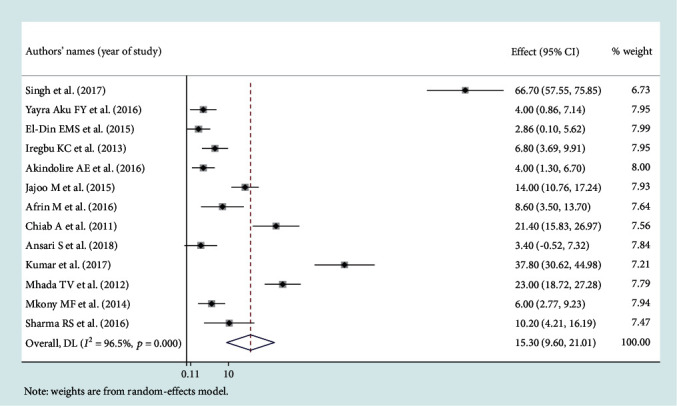
Forest plot for *E. coli* pathogen.

**Figure 6:**
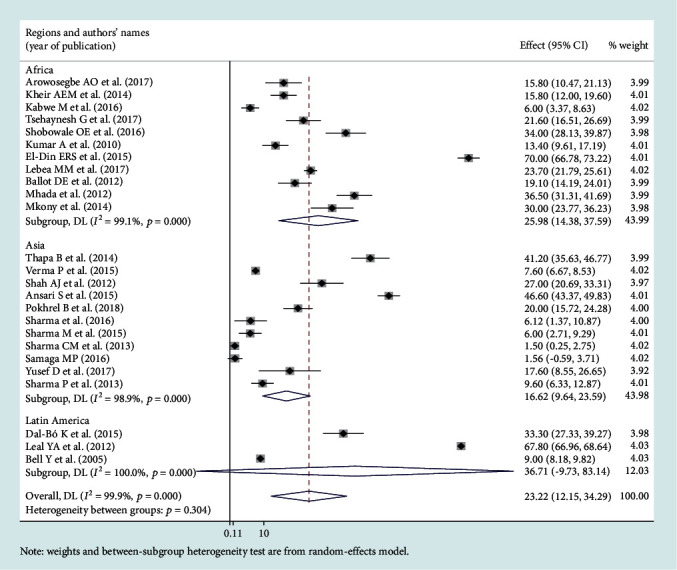
Subgroup analysis for coagulase-negative *Staphylococcus* by region.

**Figure 7:**
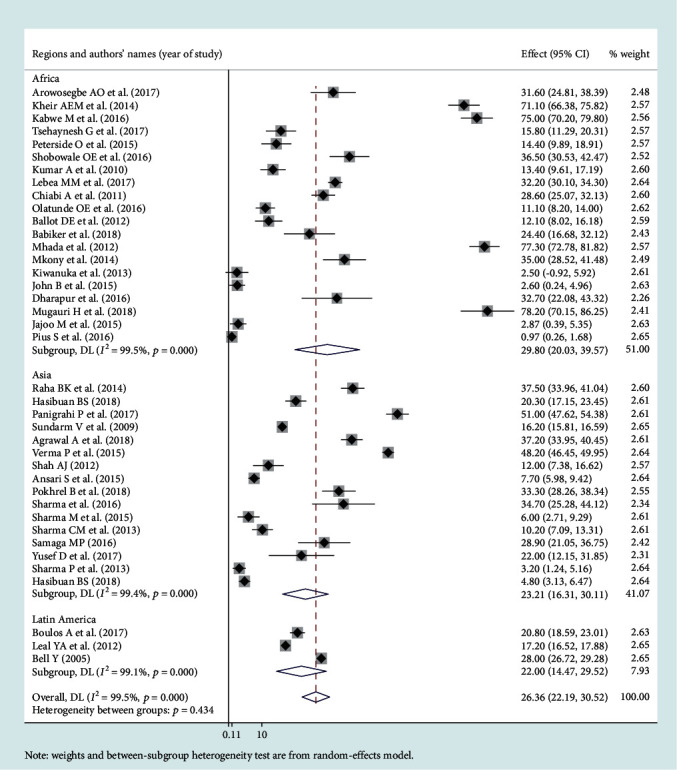
Subgroup analysis for *Klebsiella* by region.

**Figure 8:**
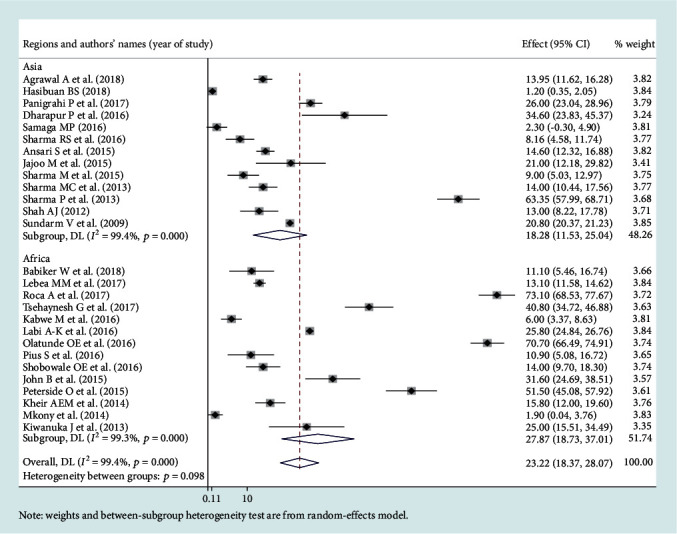
Subgroup analysis for *Staphylococcus aureus* pathogen by region.

**Figure 9:**
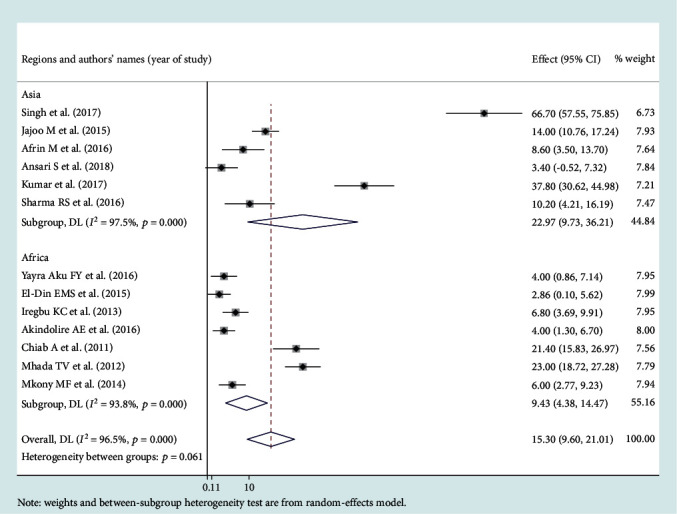
Subgroup analysis for *E. coli* pathogen by region.

Among the bacterial pathogens causing neonatal sepsis, overall pooled isolation of *CoNS* was accounted 23.22% (95% CI: 12.15-34.29) (Figure 2) and *Klebsiella* was the most prevalent causative pathogen for neonatal sepsis that accounted 26.36% (95% CI: 21.19-30.50) (Figure 3). *Staphylococcus aureus* was 23.22% (95% CI: 18.37-28.07) (Figure 4) and *E. coli* at 15.30% (95% CI: 9.60-21.01) (Figure 5). Pooled isolation of *CoNS* across continents was varied, i.e., 25.73% in Africa, 15.59% in Asia, and 36.55% in Latin America. Pooled isolation of *Klebsiella* was 31.15% in Africa, 22.98% in Asia, and 21.81% in Latin America. Pooled isolation of *Staphylococcus aureus* was 27.63% in Africa and 18.01% in Asia. Pooled isolation of *E. coli* was 22.97% in Asia and 9.43% in Africa (Figures 6–9).

### 3.4. Level of Heterogeneity

Significant heterogeneity was observed across studies of this systematic review and meta-analysis. The significant level of statistical heterogeneity across studies was assessed using the *I*^2^ test, and the presence of heterogeneity was determined through Cochran's *Q* test. A *P* < 0.05 was considered statistically significant. The overall *I*^2^ was 99.9% and belongs to *CoNS*, *P* < 0001. In Africa, Asia, and Latin America, each had overall *I*^2^ of 99.1%, 98.9%, and 100%, respectively (Figure 6). In the case of the *Klebsiella* pathogen, subgroup analysis showed that *I*^2^ = 99.5%, 99.4%, and 99.1% in Africa, Asia, and Latin America, respectively (Figure 7). *Staphylococcus aureus* also showed a significant heterogeneity with an overall *I*^2^ of 99. 4%. In subgroup analysis, Africa and Asia had an *I*^2^ of 99.3% and 99.4%, respectively (Figure 8). And also, the overall *I*^2^ for the *E. coli* pathogen was 96.5%. In subgroup analysis, Africa and Asia had an *I*^2^ of 93.8% and 97.5%, respectively (Figure 9).

### 3.5. Publication Bias

**Figure 10:**
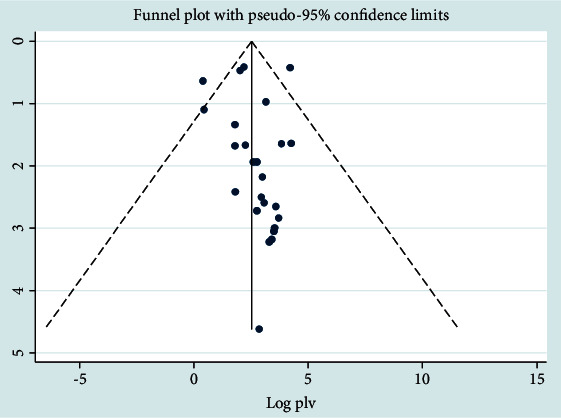
Funnel plot analysis for *CoNS* pathogen.

In the case of *CoNS*, there was no significant publication bias (*P* = 0.365), and Egger's test also had no had publication bias (95% CI, -0.7202, 0.877). The funnel plot with pseudo-95% CI using a random effect model was significantly asymmetric. The funnel plots were distributed asymmetrically at which more plots were distributed towards the right side of the midline of the graph (Figure 10). The metatrim test at pseudo-95% CI with a random effects model showed that there was no significant difference from the original pooled prevalence.

**Figure 11:**
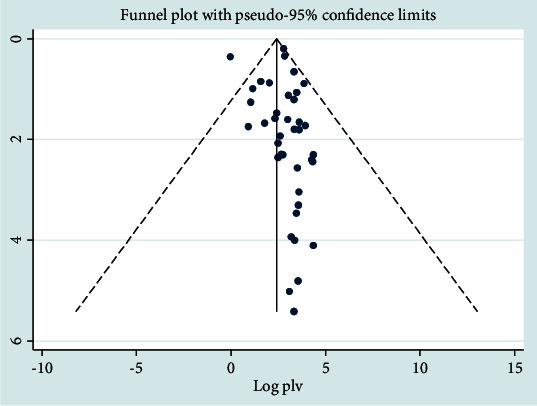
Funnel plot analysis for *Klebsiella* pathogen.

In the case of the *Klebsiella* pathogen, Begg's test and Egger's test showed that there was no significant publication bias across studies (*P* > 0.05). The number of studies missing from a meta-analysis was estimated using the trim and fill method, and there was a significant asymmetric observation. The funnel and filled funnel plot of the pseudo-95% CI showed that there was a significant asymmetric observation. This showed the presence of publication bias because we observed an asymmetric distribution of plots towards the right side of the midline with a random effects model. The model showed that there was symmetry at the top, but it was missing in the middle and bottom of the graph, and the direction of the effect is towards the right then near the bottom of the plot; we also observed a gap on the left (Figure 11). The metatrim test at pseudo-95% CI with a random effects model showed that there was no significant difference from the original pooled prevalence.

**Figure 12:**
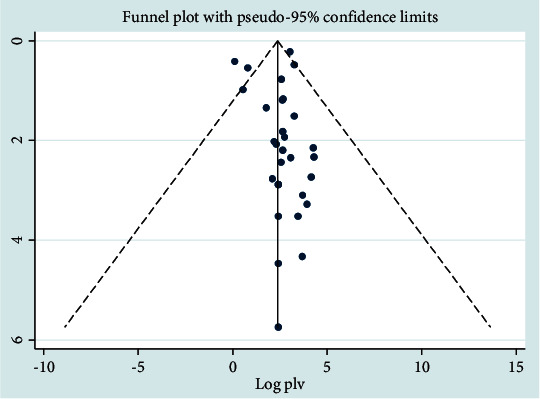
Funnel plot analysis for *Staphylococcus aureus* pathogen.

In the case of *Staphylococcus aureus*, Begg's test was applied to determine the publication bias, and statistically, there was no publication bias across studies (*P* > 0.05). Egger's test also showed no publication bias (*P* > 0.05). The distribution of plots was asymmetrical with the random effects model, and the majority of the plots were distributed towards the right side (Figure 12).

**Figure 13:**
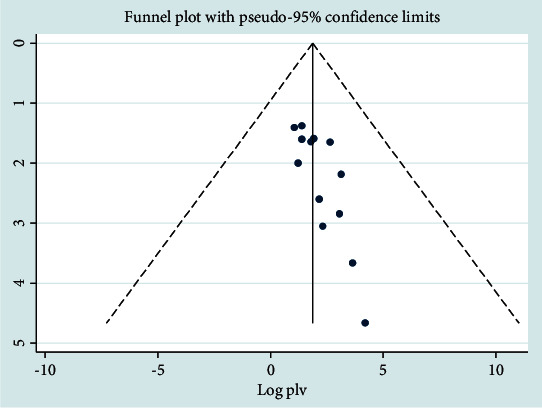
Funnel plot for *E. coli* pathogen.

In the case of *E. coli*, the funnel plot showed that there is an asymmetric distribution of plots (Figure 13), and Begg's test showed that there is a significant publication bias. However, Egger's test showed that there was no significant publication bias.

### 3.6. Metaregression Analysis

We conducted a metaregression analysis since there was statistically significant heterogeneity across the studies, with *I*^2^ test statistics less than 0.05. This analysis is vital to identify the source of heterogeneity; consequently, a corrective measure during the interpretation of findings was made. In this metaregression analysis, the study that we conducted showed that countries and cross-sectional study designs were found to be a significant source of heterogeneity. However, sample size, publication year, study quality score, and subregion were not found significant for the source of heterogeneity (Table 5).

## 4. Discussion

This review was conducted to estimate the pooled isolated common cause of neonatal sepsis in developing countries. In this study, the first most prevalent bacterial pathogen for neonatal sepsis is *Klebsiella pneumonia*, and the second most prevalent identified pathogen is *Staphylococcus aureus*. The third and fourth common pathogens are *coagulase-negative staphylococci* and *Escherichia coli*, respectively. Another systematic review and meta-analysis study on causative pathogens of neonatal sepsis in low- and middle-income countries demonstrated that the most prevalent bacterial pathogens for neonatal sepsis were *Staphylococcus aureus*, *E. coli*, and *Klebsiella* [44–46]. However, in this study, common pathogens across regions are varied in its proportion. In Africa, *Staphylococcus aureus* (27.87%) and *Klebsiella* (29.80%) were common causes of neonatal sepsis. *Coagulase-negative Staphylococcus* and *E. coli* are more common in Latin America and Asia, respectively. Similarly, *Staphylococcus aureus* and *Streptococcuspneumoniae* were most prevalent in Africa while *Klebsiella* was highly prevalent in South-East Asia [44]. Bacterial infection was a leading cause of neonatal mortality in low-income countries, and to date, it is a major cause of morbidity and mortality globally, particularly more common in developing countries as compared to developed countries [35, 47, 48] and little progress was noticed [5].

This systematic review and meta-analysis revealed that *Klebsiella pneumonia*, *Staphylococcus aureus*, and *CoNS* are the common causes of neonatal sepsis in developing countries. Similarly, a large-scale survey across the world showed that neonatal sepsis is more common in developing countries than in developed countries, and the causative pathogens were different with a predominance of Gram-negative bacteria and *Staphylococcus aureus* [35, 49, 50]. The gram-negative organism *Klebsiella* [49, 50] and the gram-positive microorganisms *Staphylococcus aureus* [50, 51] and *CoNS* [52–54] were the most common pathogens of neonatal sepsis in developing countries.

The present study demonstrated that *CoNS* is ranked as the third common cause of neonatal sepsis with a pooled prevalence of 23.22%. However, in developed countries, *Group B Streptococcus* and *CoNS* were the major organisms implicated in early-onset and late-onset sepsis, respectively [53–56], because the risk factors associated with pathogen-specific sepsis are different based on the pathogen [57].

The present study showed that there are pathogen variations across the study setting. For instance, *Klebsiella* and *Staphylococcus aureus* are the most common pathogens of neonatal sepsis in Africa, *Coagulase-negative Staphylococcus* is the predominant pathogen of neonatal sepsis in Latin America, and *E. coli* is the most common pathogen in Asia. Likewise, another systematic review showed that *Klebsiella* and *Staphylococcus aureus* represented 25% and 18% of neonatal sepsis [18]. Other systematic review findings show that *Klebsiella* is more prevalent as compared to our study, which accounted for 39% to 70% [19]. On the contrary, a systematic review of community-acquired neonatal sepsis revealed that *Staphylococcus aureus* represented 14.9% which was lower than that in our study [44] and the reason for this discrepancy may be because the pathogens causing neonatal sepsis in the communities are different from those in the health facilities and the bacterial spectrum of neonatal sepsis varies among healthcare settings and communities [35, 57].

About 70% of the cases of early-onset neonatal sepsis in the developed countries are represented by *Streptococcus agalactiae* and *Escherichia coli* [20, 58]. The majority of LOS (70%) in the developed world is due to Gram-positive infections [26, 58], *Staphylococcus aureus*, *Enterococcus* spp., and GBS, being most common in very low birth weight and preterm infants [26]. The fact is that in low-income countries, *CoNS* is responsible for the colonization and development of infection especially in low birth weight babies. Exposures to environmental risks and the timing of exposure, access to healthcare, catheter complications, immune status of the infant, and virulence of the causative agent influence the clinical expression of neonatal sepsis [59–61].

This study found that it is important to choose appropriate antibiotics based on the leading common pathogens especially for clinicians who are working in areas where there is a lack of standard laboratory methods such as blood culture. Also, in this study, it may be important to design appropriate protective measures against known germs associated with neonatal infections. It can play an important role in developing better prevention and treatment policies and programs for policymakers and health planners in low-income settings. Since there is a lack of clarity on variations in the distribution of common pathogens of neonatal sepsis in developing and developed countries [35, 49, 50], it can therefore be used as a starting point for future researchers on this topic.

A limitation of this study is a lack of data to display the community-based neonatal sepsis in African, Asian, and Latin American regions. It has also been indicated by a previous study that data on community-acquired neonatal sepsis are limited [44]. This study is also limited to protocol registration and publication. Future research should be focused on systematic review and meta-analysis of randomized clinical trial studies, and pathogen variations across settings may be the other future research area.

## 5. Conclusions

This study highlights that Klebsiella is the leading cause of the pathogen, while Staphylococcus aureus and coagulase-negative staphylococcus are the second leading cause of neonatal sepsis. E. coli is ranked as the least common cause of neonatal sepsis. This systematic review shows that these pathogens are highly prevalent in developing countries compared to the developed world. The pooled prevalence of *Klebsiella* and *Staphylococcus aureus* pathogens was predominantly high in Africa compared to other Asian and Latin American countries. *Coagulase-negative Staphylococcus* was also more prevalent in Latin America as compared to other regions. *Escherichia coli* was also more dominant in Asia compared to Africa and Latin America. Heterogeneity was identified across the regions and within each region. Since most low-income countries have no laboratory access, this finding helps in the selection of antibiotics for the empirical treatment of neonates with a high risk of sepsis. Governments should take preventive and control measures to reduce the burden of infections due to these pathogens.

## Figures and Tables

**Table 1:** Studies characteristics of *CoNS* in developing countries.

Authors' names	Years	Countries	Region	Setting	Study design	Sample size
*Africa*						
Arowosegbe et al. [62]	2017	Nigeria	Africa	Hospital	Cross-sectional	180
Kheir et al. [63]	2014	Sudan	Africa	Hospital	Cross-sectional	354
Kabwe et al. [64]	2016	Zambia	Africa	Hospital	Cross-sectional	313
Tsehaynesh et al. [60]	2017	Ethiopia	Africa	Hospital	Cross-sectional	251
Shobowale et al. [65]	2016	Nigeria	Africa	Hospital	Cross-sectional	250
Kumar et al. [66]	2010	Kenya	Africa	Hospital	Cross-sectional	310
El-Din et al. [67]	2015	Egypt	Africa	Hospital	Retrospective cohort	778
Lebea et al. [68]	2017	South Africa	Africa	Hospital	Retrospective cohort	1903
Ballot et al. [69]	2012	South Africa	Africa	Hospital	Retrospective cohort	246
Mhada et al. [70]	2012	Tanzania	Africa	Hospital	Cross-sectional	330
Mkony et al. [71]	2014	Tanzania	Africa	Hospital	Cross-sectional	208
*Asia*						
Thapa et al. [72]	2014	Nepal	Asia	Hospital	Cross-sectional	300
Verma P et al. [73]	2015	India	Asia	Hospital	Prospective cohort	3130
Shah AJ et al. [74]	2012	India	Asia	Hospital	Prospective cohort	190
Ansari et al. [75]	2015	Nepal	Asia	Hospital	Cross-sectional	918
Pokhrel et al. [76]	2018	Nepal	Asia	Hospital	Retrospective cohort	336
Sharma RS et al. [77]	2016	India	Asia	Hospital	Retrospective cohort	98
Sharma M et al. [78]	2015	India	Asia	Hospital	Prospective cohort	200
Sharma CM et al. [79]	2013	India	Asia	Hospital	Prospective cohort	364
Samaga MP [80]	2016	India	Asia	Hospital	Prospective cohort	128
Yusef D et al. [81]	2017	Jordan	Asia	Hospital	Retrospective cohort	68
Sharma P et al. [82]	2013	India	Asia	Hospital	Retrospective cohort	311
*Latin America*						
Dal-Bó et al. [83]	2015	Brazil	Latin A	Hospital	Retrospective cohort	239
Leal et al. [84]	2012	Mexico	Latin A	Hospital	Retrospective cohort	11,790
Bell et al. [85]	2005	Jamaica	Latin A	Hospital	Retrospective cohort	4702

**Table 2:** Studies characteristics of *Klebsiella* in developing countries.

Authors' names	Years	Countries	Setting	Study design	Sample size
*Africa*					
Arowosegbe et al. [62]	2017	Nigeria	Hospital	Cross-sectional	180
Kheir et al. [63]	2014	Sudan	Hospital	Cross-sectional	354
Kabwe et al. [64]	2016	Zambia	Hospital	Cross-sectional	313
Moges et al. [60]	2017	Ethiopia	Hospital	Cross-sectional	251
Peterside et al. [86]	2015	Nigeria	Hospital	Retrospective cohort	233
Shobowale et al. [65]	2016	Nigeria	Hospital	Cross-sectional	250
Kumar et al. [66]	2010	Kenya	Hospital	Cross-sectional	310
Lebea et al. [68]	2017	South Africa	Hospital	Retrospective cohort	1903
Chiabi et al. [87]	2011	Cameron	Hospital	Retrospective cohort	628
Olatunde et al. [88]	2016	Nigeria	Hospital	Retrospective cohort	450
Ballot et al. [69]	2012	South Africa	Hospital	Retrospective cohort	246
Babiker et al. [89]	2018	Sudan	Hospital	Cross-sectional	119
Mhada et al. [70]	2012	Tanzania	Hospital	Cross-sectional	330
Mkony et al. [71]	2014	Tanzania	Hospital	Cross-sectional	208
Kiwanuka et al. [90]	2013	Uganda	Hospital	Cross-sectional	80
John B et al. [91]	2015	Uganda	Health center	Cross-sectional	174
Mugauri H et al. [92]	2018	Zimbabwe	Hospital	Prospective cohort	641
Pius S et al. [93]	2016	Nigeria	Hospital	Cross-sectional	723
*Asia*					
Raha et al. [94]	2014	Bangladesh	Hospital	Cross-sectional	720
Hasibuan [95]	2018	Indonesia	Hospital	Cross-sectional	626
Jajoo M et al. [96]	2015	India	Hospital	Prospective cohort	174
Panigrahi et al. [97]	2017	India	Community	Prospective cohort	842
Sundaram et al. [11]	2009	India	Hospital	Retrospective cohort	34362
Agrawal et al. [98]	2018	India	Hospital	Cross-sectional	850
Verma et al. [73]	2015	India	Hospital	Prospective cohort	3130
Shah et al. [74]	2012	India	Hospital	Prospective cohort	190
Dharapur et al. [99]	2016	India	Hospital	Cross-sectional	75
Ansari et al. [75]	2015	Nepal	Hospital	Cross-sectional	918
Pokhrel et al. [76]	2018	Nepal	Hospital	Retrospective cohort	336
Sharma et al. [77]	2016	India	Hospital	Retrospective cohort	98
Sharma M et al. [78]	2015	India	Hospital	Retrospective cohort	200
Sharma CM et al. [79]	2013	India	Hospital	Retrospective cohort	364
Samaga MP [80]	2016	India	Hospital	Retrospective cohort	128
Yusef D et al. [81]	2017	Jordan	Hospital	Retrospective cohort	68
Sharma P et al. [82]	2013	India	Hospital	Retrospective cohort	311
Hasibuan BS [95]	2018	Indonesia	Hospital	Cross-sectional	626
*Latin America*					
Boulos et al. [100]	2017	Haiti	Hospital	Retrospective cohort	1292
Leal et al. [84]	2012	Mexico	Hospital	Retrospective cohort	11,790
Bell et al. [85]	2005	Jamaica	Hospital	Retrospective cohort	4702

**Table 3:** Study characteristics of *Staphylococcus aureus* in developing countries.

Authors' names	Years	Countries	Region	Setting	Study design	Sample size
*Asia*						
Agrawal A et al. [98]	2018	India	Asia	Hospital	Cross-sectional	850
Hasibuan BS [95]	2018	Indonesia	Asia	Hospital	Cross-sectional	626
Panigrahi P et al. [97]	2017	India	Asia	Community	Prospective cohort	842
Dharapur et al. [99]	2016	India	Asia	Hospital	Cross-sectional	75
Samaga MP [80]	2016	India	Asia	Hospital	Prospective cohort	128
Sharma RS et al. [77]	2016	India	Asia	Hospital	Retrospective cohort	98
Ansari S et al. [75]	2015	Nepal	Asia	Hospital	Cross-sectional	918
Jajoo M et al. [96]	2015	India	Asia	Hospital	Prospective cohort	174
Sharma M et al. [78]	2015	India	Asia	Hospital	Prospective cohort	200
Sharma CM et al. [79]	2013	India	Asia	Hospital	Prospective cohort	364
Sharma P et al. [82]	2013	India	Asia	Hospital	Retrospective cohort	311
Shah AJ [74]	2012	India	Asia	Hospital	Retrospective cohort	190
Sundaram V et al. [11]	2009	India	Asia	Hospital	Retrospective cohort	34362
*Africa*						
Babiker W et al. [89]	2018	Sudan	Africa	Hospital	Cross-sectional	119
Lebea MM et al. [68]	2017	South Africa	Africa	Hospital	Retrospective cohort	1903
Roca A et al. [101]	2017	Gambia	Africa	Health Center	Retrospective cohort	361
Tsehaynesh G et al. [60]	2017	Ethiopia	Africa	Hospital	Cross-sectional	251
Kabwe M et al. [64]	2016	Zambia	Africa	Hospital	Cross-sectional	313
Labi A-K et al. [102]	2016	Ghana	Africa	Hospital	Retrospective cohort	8025
Olatunde OE et al. [88]	2016	Nigeria	Africa	Hospital	Prospective cohort	450
Pius S et al. [93]	2016	Nigeria	Africa	Hospital	Cross-sectional	723
Shobowale OE et al. [65]	2016	Nigeria	Africa	Hospital	Cross-sectional	250
John B et al. [91]	2015	Uganda	Africa	Health center	Cross-sectional	174
Peterside O et al. [86]	2015	Nigeria	Africa	Hospital	Retrospective cohort	233
Kheir et al. [63]	2014	Sudan	Africa	Hospital	Cross-sectional	354
Mkony et al. [71]	2014	Tanzania	Africa	Hospital	Cross-sectional	208
Kiwanuka et al. [90]	2013	Uganda	Africa	Hospital	Cross-sectional	80

**Table 4:** Studies characteristics of *E. coli* in developing countries.

Authors' names	Years	Countries	Region	Setting	Study design	Sample size
Singh et al. [103]	2017	India	Asia	Hospital	Retrospective cohort	102
Aku FY et al. [104]	2016	Ghana	Africa	Hospital	Cross-sectional	150
El-Din EMS et al. [67]	2015	Egypt	Africa	Hospital	Cross-sectional	140
Iregbu KC et al. [105]	2013	Nigeria	Africa	Hospital	Retrospective cohort	251
Akindolire AE et al. [106]	2016	Nigeria	Africa	Hospital	Cross-sectional	202
Jajoo M et al. [96]	2015	India	Asia	Hospital	Cross-sectional	440
Afrin M et al. [107]	2016	Bangladesh	Asia	Hospital	Cross-sectional	116
Chiabi A et al. [87]	2011	Cameroon	Africa	Hospital	Cross-sectional	208
Ansari S et al. [75]	2018	Nepal	Asia	Hospital	Cross-sectional	82
Kumar et al. [108]	2017	India	Asia	Hospital	Cross-sectional	175
Mhada TV et al. [70]	2012	Tanzania	Africa	Hospital	Cross-sectional	371
Mkony MF et al. [71]	2014	Tanzania	Africa	Hospital	Cross-sectional	208
Sharma RS et al. [77]	2016	India	Asia	Hospital	Retrospective cohort	98

**Table 5:** Metaregression analysis of *Coagulase-negative Staphylococcus*, *Staphylococcus aureus*, and *E. coli* in developing countries.

Variable	Coefficient	*P* value
*Coagulase-negative Staphylococcus*		
Years of study	-0.17	0.826
Sample size	-0.23	0.983
Brazil	-35.24	0.027
Ethiopia	-70.95	0.002
India	-61.44	0.000
Jamaica	-59.55	0.001
Jordan	-50.94	0.005
Kenya	-79.15	0.001
Mexico	-0.745	0.956
Nepal	-48.54	0.005
Nigeria	-67.75	0.002
South Africa	-47.81	0.001
Sudan	-76.75	0.001
Tanzania	59.23	0.004
Zambia	-86.55	0.001
Cross-sectional	24.00	0.044
Prospective	1.45	0.813
*Staphylococcus aureus*		
Years of study	-0.48	0.483
Sample size	-0.23	0.654
Ethiopia	49.42	0.134
Gambia	60.00	0.046
Ghana	22.20	0.472
India	18.84	0.406
Indonesia	9.74	0.758
Nepal	23.22	0.466
Nigeria	34.65	0.163
Sudan	21.30	0.437
Tanzania	18.62	0.558
Uganda	34.48	0.236
Zambia	14.62	0.644
Cross-sectional	-21.72	0.149
Prospective cohort	-9.50	0.445
*E. coli*		
Year of study	-0.6604672	0.556
Sample size	0.022806	0.401
Bangladesh	5.2	0.866
Cameroon	18	0.565
Egypt	-0.5400002	0.986
Ghana	0.5999999	0.984
India	28.44278	0.273
Nigeria	1.998955	0.940
Tanzania	11.07911	0.679

## Abbreviations

CoNS: Coagulase-negative *Staphylococcus*
*E. coli*: *Escherichia coli*
EONS: Early-onset neonatal sepsis
LOS: Late-onset sepsis.

## Supplementary Materials

Supplementary Materials This section contains the methods how to search for articles in the PubMed/Medline databases. The search was conducted by searching Medical Literature Using Medical Subject Headings (MeSH) terms. These were neonate OR neonatal OR infant OR newborn AND pathogens OR coagulase-negative staphylococcus OR *Staphylococcus aureus* OR *Klebsiella* OR *Escherichia coli* AND developing countries OR developing country OR countries OR developing OR least developed countries OR least developed country OR less developed countries OR nations OR under developed OR under developed nations OR third world countries OR third world country OR third world nations. This information can be found in the last part of this manuscript appended in the annex.
