# Supplementary material for: A Systemic Review and Meta-analysis of the Leading Pathogens Causing Neonatal Sepsis in Developing Countries
Source: Biomed Res Int. 2021 Jun 5;2021:6626983. doi: 10.1155/2021/6626983 (PMC8203353; doi:10.1155/2021/6626983)
Supplement: Supplementary Materials — This section contains the methods how to search for articles in the PubMed/Medline databases. The search was conducted by searching Medical Literature Using Medical Subject Headings (MeSH) terms. These were neonate OR neonatal OR infant OR newborn AND pathogens OR coagulase-negative staphylococcus OR Staphylococcus aureus OR Klebsiella OR Escherichia coli AND developing countries OR developing country OR countries OR developing OR least developed countries OR least developed country OR less developed countries OR nations OR under developed OR under developed nations OR third world countries OR third world country OR third world nations. This information can be found in the last part of this manuscript appended in the annex. [file 6626983.f1.docx]

**Supplementary file**

**Search terms**

(((((((((((((("pathogens"[All Fields] AND (causative[All Fields] AND agent[All Fields])) AND (("coagulase"[MeSH Terms] OR "coagulase"[All Fields]) AND negative[All Fields] AND ("staphylococcus"[MeSH Terms] OR "staphylococcus"[All Fields]))) AND ("klebsiella"[MeSH Terms] OR "klebsiella"[All Fields])) AND ("escherichia coli"[MeSH Terms] OR ("escherichia"[All Fields] AND "coli"[All Fields]) OR "escherichia coli"[All Fields])) AND ("staphylococcus aureus"[MeSH Terms] OR ("staphylococcus"[All Fields] AND "aureus"[All Fields]) OR "staphylococcus aureus"[All Fields] OR ("staph"[All Fields] AND "aureus"[All Fields]) OR "staph aureus"[All Fields])) AND ("infant, newborn"[MeSH Terms] OR ("infant"[All Fields] AND "newborn"[All Fields]) OR "newborn infant"[All Fields] OR "neonate"[All Fields])) OR "infant, newborn"[MeSH Terms]) OR "infant"[MeSH Terms]) OR "infant, newborn"[MeSH Terms]) OR ("sepsis"[MeSH Terms] OR "sepsis"[All Fields])) AND ("infections"[MeSH Terms] OR "infections"[All Fields])) AND ("developing countries"[MeSH Terms] OR ("developing"[All Fields] AND "countries"[All Fields]) OR "developing countries"[All Fields])) AND ("africa"[MeSH Terms] OR "africa"[All Fields])) AND ("latin america"[MeSH Terms] OR ("latin"[All Fields] AND "america"[All Fields]) OR "latin america"[All Fields])) AND ("asia"[MeSH Terms] OR "asia"[All Fields]).
